# The Aesthetic Form of Perezhivanie: An Ethnographic Study of Becoming an Environmental Scholar-Activist

**DOI:** 10.1007/s42087-026-00682-3

**Published:** 2026-07-22

**Authors:** Sohrab Rezvani

**Affiliations:** Manchester Institute of Education, Manchester, UK

**Keywords:** Perezhivanie, Personality, Environmentalism, Bakhtin, Scholar-Activism

## Abstract

*Perezhivanie* as one of the main concepts of Cultural Historical Activity Theory (CHAT), related to psychology and personality development, has been widely used by scholars interested in pedagogy, teacher training and language learning. However, multiple interpretations and vagueness of the concept have made its meaning too elusive. This article a theoretical contribution to further development of this category through enriching it with Bakhtin’s aesthetic theory, informed by autoethnographic material. Bakhtin’s theory of artistic creation can provide analytical tools to understand the creative process of deep changes in the psychological system through an aesthetic lens and conceptualise it via literary devices. In this article I propose three situations (absurd, picaresque, and ressentiment) as examples of how the dialogue between CHAT and Bakhtin’s aesthetic theory can provide insight in understanding deep personality change and development. In the end, I have used the theoretical framework as an interpretive tool to briefly analyse the autoethnographic data in relation to my own situation of becoming an environmental scholar-activist.

## Introduction

*Perezhivanie*, one of the main concepts in Cultural Historical Activity Theory (CHAT), has recently received great attention among scholars who are interested in pedagogy, teacher training and identity formation, personality development, language learning, and other subjects related to psychology and education (for example, see the special issue of the journal of Mind, Culture and Activity *Symposium on Perezhivanie*, Vol.24, Iss.4). Broadly, perezhivanie is understood to be the process of emotional experiencing and making sense of reality. However, as Versov and Fleer (2016) observed, understanding of this concept remains elusive. Smagorinsky (2011) argues that “*Perezhivanie* thus far remains a tantalizing notion than a concept with clear meaning”. Gonzalez Rey writes that there are multiple interpretations of perezhivanie because “Vygotsky never offered an ontological definition of the concept” (González Rey, 2016, p. 306), and himself used the concept differently across his work. There is also little agreement among various scholars about how to translate the word into English (Cole & Gajdamschko, 2016).

Clara (2016) argues that the semantic richness and multiple connotations of perezhivanie were useful in the infancy period of this concept, since they allowed the concept to find many lives in different lines of research. However, continuous fogginess around the meaning of perezhivanie hinders its potential for development in the future. Having a clearer conception of perezhivanie is necessary to establish it as a valid scientific concept. In this article I will contribute to the development of the concept by mapping various interrelated psychological processes that have been named perezhivanie (or moments of a perezhivanie). Moreover I will contribute to further development of our understanding of one psychological process within the cluster of perezhivanie processes: ‘perezhivanie-as-struggle’ (Vasilyuk, 1986, Clara, 2016 b) by highlighting the potential offered through cross-fertilization with aesthetic theories. ‘Perezhivanie-as-struggle’ is a process of radical changes and ruptures in the consciousness and personality, in such a way that leads into re-organisation of the whole psychological system that interacts with the environment (Clara, 2016). I argue here that one can examine these radical changes and ruptures using literary devices and artistic, aesthetic theories that have been produced to study literature and works of art.

This paper argues that since perezhivanie-as-struggle is a creative process, it is possible to study the process and products of that creation (new consciousness and new personality) in this way. I will examine the aesthetic form of the process of ‘perezhivanie-as-struggle’ through autoethnographic research on becoming an environmental scholar-activist, highlighting the significance of such knowledge for developing educational tools to develop the character of sensibilities of activists. In this paper I mainly incorporate Bakhtin’s aesthetic theory because of the shared ideas and principles as well as existing tradition of dialogue between Vygotskian and Bakhtinian scholars (for example look at Holland, 2001; Williams & Ryan, 2019; and Rosborough, 2016). Brandist (2007) observes several similarities between works of Bakhtin and Vygotsky including “the centrality of dialogue to the development of human consciousness, the understanding of inner speech as internalised dialogue and the emergence of culture from primordial mythical thinking to mention but three” (p79). These similarities, as well as their shared interest in doing inquiries on the psychology of aesthetic activity rooted in traditions of dialectics and Marxism makes it a sensible choice to bring Bakhtin’s theory into further dialogue with Vygotskian concept of perezhivanie-as-struggle.

## Perezhivanie

The term perezhivanie itself originates from the Russian verb “perezhivat” “Zhivat means “to live” and pere means carrying something over something, letting something pass beneath and overleaping it…” (Blunden, 2016, p. 276). This word has an everyday meaning in Russian people’s common sense with connotations around “experience”, “feeling”, “living through” strong tensions, anxieties, or other emotional experiences. It also has a significant place in Russian literature and art. Tolstoy described the proper activity of art to be the expression of felt experiences so that others would experience (*perezhivayut*) the same emotions (Tolstoy, 1995). Stanislavsky’s theatrical system introduced perezhivanie as a form of theatre where the actor re-lives a past experience related to the one they are trying to express, instead of simply representing the character through an inauthentic imitation. This meant that for Stanislavsky, perezhivanie was concerned with the internal psychological side of performance, or the inner theatrical sense of self that the actor is experiencing, rather than the outer theatrical sense of self that the actor is embodying in his performance. To allow actors to practice authentic method acting, he developed a set of tools. (Carnicke, 2009; Pitches, 2005).

Whilst Tolstoy and Stanislavsky were concerned with aesthetic experience, Vygotsky argued that aesthetic experience provides a clue to our understanding of experience in general. This was because, for Vygotsky, experience itself contained an aesthetic premise. In his first major work, *Psychology of Art*, he explores the psychology of aesthetic reactions and its relation to emotional experience. Vygotsky tried to study aesthetic reactions to works of art (in his cases, fables, novels and tragedies). Drawing on Aristotle’s theory of catharsis, he argues that aesthetic reaction not only reflects contradictory emotions but also brings about changes and reorganisations in our emotions. As Gonzales Rey (2016) notes aesthetic reaction, at the core of perezhivanie, is incomprehensible and hidden from the subject herself - it is an unconscious process, and its immediate nature is inaccessible. However, it is possible to observe the expressions of *aesthetic reaction* through the physical response of the subject. Two years before his death, Vygotsky returned to the psychology of aesthetic reaction and perezhivanie of actors in ‘On the Problem of the Psychology of the Actor’s Creative Work’ (Vygotsky, 1987, pp. 237–244) where he emphasises that:

The psychology of the actor is a historical and class category, not a biological category. The idea central to all new investigations that determines the approach to the concrete psychology of the actor is expressed in this one aspect alone. Consequently it is not biological patterns primarily that determine the character of the actor’s stage perezhivaniya. These perezhivaniya comprise a part of the complex function of the artistic work that has a definite social, class function historically established by the whole state of the mental development of the epoch and class, and, consequently, the laws of coupling passions and the laws of interpreting and interweaving feelings of a role with feelings of the actor must be resolved primarily on the historical plane and not on the plane of naturalistic (biological) psychology. Only after this resolution can the question arise of how, from the point of view of the biological patterns of the mind, one or another historical form of the actor’s work is possible.

Moreover, Perezhivanie for Vygotsky is an unconscious subjective process that takes place in psychological systems that take us through awareness, emotional reaction, interpretation and response. Vygotsky defines psychological systems as “flexible, historically developing systemic relationships between psychological functions” (Dafermos, 2018, p.158). These systems are comprised of multiple mental functions, some which are “lower” or innate, biologically determined mental functions, such as basic perception, reflexes, and natural memories, and some of which are “higher” or historically developed mental functions such as logical reasoning, abstract thinking, voluntary attention and volition all of which are developing through working with specific ideas, concepts, images, and other cultural tools.

Each psychological system acts as a prism (Vygotsky, 1994, pp. 338–354) for refraction of the environment, enabling the individual to relate to it and giving the potential to act in it. For Cong-Lem, there is a two-stage process, whereby “*interpretation* refers to how the individual makes sense of the current event, whereas *generalisation* indicates their capacity to understand the true meaning and significance of the event beyond the current event/experience” (Cong-Lem, 2022, p233). These psychological systems or prisms make sense of *the situation as a whole* “including all relevant aspects of the external situation and all relevant aspects of the subject facing that situation” (Clara, 2016, p.5). As Vygotsky put it:

A perezhivanie is a unit where, on the one hand, in an indivisible state, the environment is represented, i.e. that which is being experienced—a perezhivanie is always related to something which is found outside the person—and on the other hand, what is represented is how I, myself, am experiencing this, i.e., all the personal characteristics and all the environmental characteristics are represented in perezhivanie; (…) So, in a perezhivanie we are always dealing with an indivisible unity of personal characteristics and situational characteristics, which are represented in perezhivanie. (Vygotsky, 1994, p343)

Perezhivanie for Vygotsky is the set of psychological processes that an individual lives through in order to emotionally relate, become aware of, interpret, and generalise ideas from situations that would provide them the consciousness to act in the environment. Perezhivanie happens through dynamic, growing, systems of mental functions (prisms), based on unconscious emotions and aesthetic reactions. One of the significant philosophical and theoretical implications of the concept of perezhivanie is that any cognitive or moral act is deeply related to unconscious emotions and aesthetic reactions to the environment. In other words, our cognitive knowledge and our ethical evaluation of reality are inherently interwoven to our unconscious emotions and aesthetic reactions. Perezhivanie makes a link between psychology, aesthetics, scientific and ethical theories.

The concept of *perezhivanie*, from a methodological point of view, can be used as an analytical tool to study individuals’ conduct in everyday life. As a unit of analysis, it provides us with a lens to observe the basic characteristics of consciousness and personality as a whole. And since the problem of development of consciousness and personality was at the core of Vygotsky’s scientific project (Dafermos, 2018, p56) I argue that the concept of *perezhivanie* is at the heart of Vygotsky’s political and scientific project to create a pedagogy to allow the *socialist alteration of the human* and the making of *all-round developed and flexible personalities* “who would be capable of changing the forms of work, and organising the production process and controlling it”. (Vygotsky, 1994, p180). For this reason, I argue that using perezhivanie without considering the political context of the whole theory of CHAT and the pedagogical relevance of it has also added to the confusion around this concept. Perezhivanie is part and parcel of a theory of struggle for socialism and domesticated conceptions of it will not be faithful to the original Vygotskyian version.

## Perezhivanie-as-struggle

As I have mentioned, perezhivanie is the cluster of processes of making sense of the totality of the environment. But what happens to individuals when they cannot make sense of the environment anymore? When their prisms cannot meaningfully and coherently refract their environment? When they become frustrated, or it becomes impossible for them to decide between conflicting inclinations? What do individuals do when they are faced with a situation of strong pain, overwhelming love, suffering, and psychological disruptions that they cannot do anything, both practically and mentally, to resolve it?

As Vasilyuk (1991) observes:

The actual character of a psychological situation is, in the last count, determined by the “internal state” created. Although a situation may be objectively insoluble, so long as the individual retains a belief in its solubility, … that situation has not become critical in the exact sense of the term. Conversely, even if the situation is from the viewpoint of an outside observer entirely resolvable, so soon as the individual acquires a conviction of its being impossible the situation then becomes a critical one, with all its attendant consequences. (p. 32)

The work of struggling with the “impossible situation” i.e., the many battles of making change in the self and environment that lead to gaining a new psychological equilibrium are also moments of *perezhivanie*. To make this moment more visible and distinguishable, following Clara’s insights on Vygotsky and Vasilyuk’s conception of *perezhivanie* (Clara, 2016), one can call this moment *perezhivanie-as-struggle* in contrast to *perezhivanie-as-contemplation*. In other words, *perezhivanie-as-contemplation* is the gradual meaning-making process of psychological systems during the times of peace and equilibrium while the activity of *perezhivanie-as-struggle* is the critical, spiky re-organisation of psychological systems and makings of new formations/neoformations during the times of war and rupture^1^.

One can find the roots of Perezhivanie-as-struggle in Vygotsky’s work when he was investigating the problem of dividing the child’s development into different periods and ages. He argues that the development of children happens through relatively firm or stable ages that development occurs mainly as “microscopic changes occurring daily in the personality of the child which accumulate to a certain point”, but this stable period of gradual accumulation leads into “a single leap, appear as a given age neoformation” (Vygotsky, 2021 p.26). For Vygotsky neoformation is a “new type of construction of the personality and its activities … which occur in a given stage for the first time and which in an important and basic way define the consciousness of the child in relation to his environment” (Vygotsky, 2021 p.25). In his final observations on the problem of periodization of child’s development, Vygotsky observes that “for every perezhivanie, there exists a real, dynamic effect of the environment in relation to the child. From this point of view, the essence of each crisis is the reconstruction of inner perezhivanie, the restructuring of which is rooted in the change of the main moment that determine the child’s relationship to the environment, precisely the changes in the child’s needs, drives, and motives of child behavior. The growth and change in needs and drives presents the least conscious and least volitional portion of the personality, and in the transition from age to age in the child, there appear new drives and new motives; in other words, the engines of his activity are undergoing a reassessment of values.” (Vygotsky, 2021 p241). In other words, what Vasilyuk has developed for the conception of the critical situations and deep personality changes in adult life is continuation of the same line of argument that Vygotsky made for the development of children.

To re-state the concept of perezhivanie as I developed it earlier and as I have illustrated it in the Fig. 1, it is a developmental process of re-creation, re-organisation and reconfiguration of the psychological system (or the prism) that refracts the totality of the environment into the individual’s consciousness and enables her to act upon the environment based on her unconscious emotions, and aesthetic reactions that provide the initial germ-cell of the unification of knowledge of reality and ethical imperatives. Based on this, the will forms itself.

**Fig. 1:**
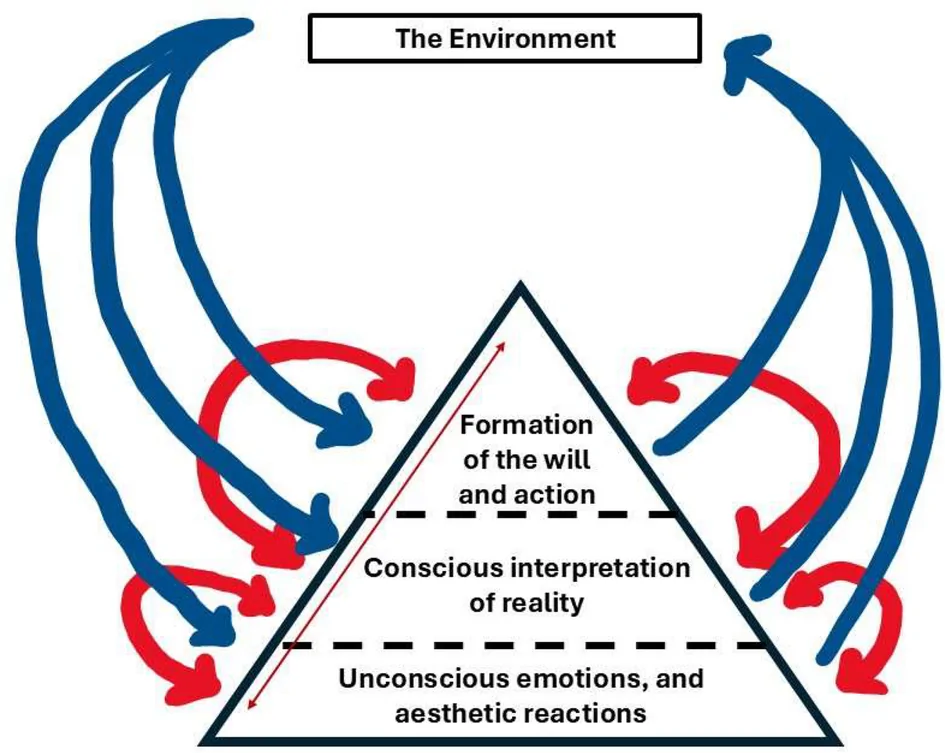
The Dialectical Relation Between the Psychological System and Environment

Vasilyuk made the theoretical framework for understanding the internal structure of *perezhivanie-as-struggle* with the objective of contributing to the field of clinical psychology and the practice of psychotherapy (Vasilyuมk, 1991). To use his framework for other purposes and in other fields, such as critical pedagogy, we need to bring other concepts and theories into play. One angle that is particularly useful for developing Vasilyuk’s framework in the context of pedagogy is aesthetic theory, which, not coincidentally, was where Vygotsky began. This allows us to understand the changes in aesthetic appreciation and intuition during the *perezhivanie-as-struggle*. It allows a focus on what happens to the person’s taste, and their way of weaving cognition and moral values into a beautiful internal relation, and how changes and shifts in the aesthetic sensibilities lead to changes in activity. While Vasilyuk in his case study of Rodion Raskolnikov^2^, is interested in the conscious elements of Raskolnikov’s drive to self-actualisation, he does not pay much (or any) attention to the unconscious elements, which, as we discussed earlier, are comprised of emotional responses and aesthetic reactions.

## Aesthetics and Perezhivanie

Since the formation of our consciousness lies primarily in our emotional responses and aesthetic sensibilities, investigation into the changes in our aesthetic sensibilities as we experience critical moments will help us develop a theory, and maybe a practice, of the pedagogy of such moments. For this reason, I now bring into theoretical dialogue the theory of Perezhivanie (Following Vygotsky and Vasilyuk) and the aesthetic theory of Mikhail Bakhtin, the Russian philosopher and literary critic. Bakhtin’s theory of aesthetics (Bakhtin, 2021) is a good choice for this dialogue and enrichment due to his specific interpretation of aesthetic creativity that integrates cognitive and moral aspects into its theory of the beautiful objects. Moreover, his theory of aesthetic form provides useful devices to analyse the aesthetic form of a perezhivanie-as-struggle.

Aesthetic activity, for Mikhail Bakhtin, is an activity of reorganising a ‘material’ into an artistic object containing ideas, emotions, desires, interpretations and the “axiological relationship of the author and the contemplator to something apart from the material” (Bakhtin, 1990, p264). For example, when a sculptor works on a marble stone to bring out the sculpture of a man, he is not only related to the ‘material’ (marble) but also to the unfolding ‘content’ and ‘form’ of the artistic object. Although that content and form express themselves within the reorganised material, they are pointing to relations beyond that material. One should not confuse the content of an aesthetic activity with the sensuous reality of a reorganised material. The content of an aesthetic activity is not the physical and mathematical information that one can grasp from a sculpture or a piece of music, but the work of the aesthetic activity (either by the artist or the contemplator) which can be perceived in the ideas, emotions, desires and axiological relationships contained in the work of art.

As Bakhtin observes: “The aesthetician must become a geometer, a physicist, an anatomist, a physiologist, a linguist, just as the artist [is] to some degree” (Bakhtin, 1990, p267). In other words, in a complete aesthetic analysis, one should understand “the aesthetic object in its purely artistic distinctiveness and its structure” (Bakhtin, 1990, p.267). The form and the content of the work of art is distinct from the external material work; the latter should be analysed as only “the technical apparatus of the aesthetic execution” (Bakhtin, 1999, p.267).

The form of the artistic object has two features. The ‘compositional form’ refers to the internal structure of the art, its style, genre, techniques, and the devices that have been used to reorganise the material. But the ‘architectonic form’ refers to the external structure of the art, the axiological relationships, the author-reader relationship, and the cultural meaning of the work of art. For example, Bakhtin explains that “*Drama* is a compositional form (dialogue, division into acts, and so on), but the *tragic* or *comic* are the architectonic forms of consummation” (Bakhtin, 1990, p.269). In other words, the ‘architectonic form’ is the *formic content* of the ‘compositional form’ of a work of art. Every architectonic form will be realised through the execution of specific compositional devices, and the essential architectonic forms realised in the organised material correspond to those compositional devices. Therefore, the compositional devices should be studied by teleological methods: To what extent they have fulfilled their task of realising an architectonic form? And to what extent do they fail to do so?

While delving into these aspects of aesthetic analysis, we should not forget that the purpose of this exploration is to appropriate concepts and theories that would serve the development of our pedagogical theory of *perezhivanie-as-struggle* as an aesthetic activity. Hence, here I am considering a person’s *perezhivanie-as-struggle* (the activity of changing psychological systems and sensibilities) as the aesthetic activity that can be analysed through the lens of aesthetic theories. For example, we can study the compositional form (the style, genre, techniques and devices that one has used to change one’s sensibilities) and the architectonic form (the ideas, emotions, desires, and axiological relationships that one is expressing) of this whole process.

What is the content of an artistic object? For Bakhtin, it is the intuitive and subjective unification of a cognitive and ethical understanding of reality; The meaning and the value. It is a constitutive moment in the formation of an artistic object, where ‘what is out there’ and ‘what is to be done’ show themselves in a unified way, as a *beautiful* thing. And the aesthetic form is “the expression of that essential relation to the world of cognition and action” (Bakhtin, 1990, p.281). It is the creative, form-giving activity of the author to bring about the unification of cognitive knowledge and an ethical imperative. Perezhivanie-as-struggle, as the aesthetic activity, contains a radical recreation and reorganisation of the relation between meaning and value in an individual.

## Ongoing Perezhivanie-as-Struggles and their Aesthetic Forms

Vasilyuk (1991), reviewed the existing psychotherapeutic literature to find out how most scholars viewed the process of ‘unsuccessful’ perezhivanie-as-struggle. Most felt that this was characterised by a distortion of reality, bringing about self-deception, and reproducing rigid, compulsive, and involuntary processes. Vasilyuk observes that unsuccessful perezhivanie-as-struggle will develop psychological defences that eventually lead into the disintegration of behaviour, regression, and the deformation of character. On the other hand, successful perezhivanie-as-struggle leads to development, self-actualization, improvement of personality, and an increase in adaptive capabilities, such as being realistic, flexible, and feeling a sense of voluntary choice.

It is worth noting that unsuccessful and successful types of perezhivanie-as-struggle are ideal types and, in reality, perezhivanie-as-struggles will create forms that have characteristics of both ideal types. Perezhivanie-as-struggle, on the one hand, is a developmental process. We broaden our environment and our understanding of it changes. We always resolve (in some way) the predicaments, escape the meaningless, impossible situations and reorganise our prism. On the other hand, within this process, sometimes we don’t achieve a developmental change (one that will improve our ability to act) but instead we go through regressive changes (one that damages our ability to act). How can aesthetic theories help us grasp this contradictory characteristic of perezhivanie-as-struggle? How can we express the moment when a person encounters predicaments that are so alarmingly incomprehensible that the person experiences one form of impossibility or another? Existing theories of ‘perezhivanie’ say very little about those moments where the constituent urges for a situation of development are present, but no development is ongoing or even conceivable. These situations can take many forms. In what follows, using aesthetic theories and literary criticism, I will examine three forms: Absurd, Picaresque and Ressentiment situations.

### Absurd Situations

What can we learn from aesthetic theories of absurd situations? In absurd situations there is “a distance or gap, however wide or narrow, that separates and sometimes degrades the artist’s impulse or will toward creation from the physical reality of the incarnation … This means that a measure of distance persists between the author’s intent or desire or will and the resultant manifestation” (Sollars, 2013). The limitations of one’s abilities and imperfection of their tools and words to bring into material form their ideals are the essence of an absurd situation. One can consider absurdity to be one of the characteristics of every situation. Because there is always, more or less, a gap between an individual’s will and the restrictions of life to realise that will. As Marx says in the beginning of *The Eighteenth Brumaire of Louis Bonaparte* “Men make their own history, but they do not make it as they please; they do not make it under self-selected circumstances, but under circumstances existing already, given and transmitted from the past.” (Marx, 2021, p9). However, one can consider situations where the dominant characteristic is absurdity. Situations that bring about purposeless, senseless and useless activities (Bennett, 2015). As Esslin observes, one of the typical characteristics of the ‘Theatre of the Absurd’ is that these plays “[tend] toward a radical devaluation of language … what happens on the stage transcends, and often contradicts, the words spoken by the characters” (Esslin, 1974, p21). There is no cohesion between the characters’ consciousness and intention and their actions in reality. In this literary genre there is “no story or plot to speak of … these are often without recognizable characters and present the audience with almost mechanical puppets … these often have neither a beginning nor end … these seem often to be reflections of dreams and nightmares … these often consist of incoherent babblings” (Esslin, 1974, p18). In some absurd situations in art, we are dealing with the ridiculous incapacity of the character in the environment that evokes derisive laughter.

Perezhivanie-as-struggle as a concept that articulates the radical change in the human character and personality, must often include a feeling of the absurd - that is the impossibility of realising one’s desires. One can observe social situations that require the reconstruction of personality while not providing the means to realise that objective. Using Bakhtin’s terminology, the architectonic form of activity in those situations are absurdity. This is, also, the situation that is unable to unify a coherent moral and cognitive articulation of reality.

### Picaresque Situations

We find another analogy in aesthetic theory when we are analysing situations where the individual finds a real impossibility of arriving at a meaning of *totality* of environment, while at the same time she feels it’s impossible to continue without such meaning. For example, it might be that the individual can make some disjointed and episodic meanings out of each project that she is involved with, but she is unable to make a meaning out of the *totality* of the environment, which is to say the totality appears meaningless. Such might be the experience, for example, of people who do very short-term jobs in various cities, engaging with various little projects, and making very short-term connections with different people, without being able to find any coherent framework to put all these episodes together. In artistic theory, picaresque novels portray such lives-without-meaning, partly through the narrative structure. Northrop Frye distinguishes two structures of narratives: *and-then* structure and *hence* structure. In *and-then* narrative structure the plot is not growing systematically, and the episodes are coming after each other in a relatively erratic or capricious way. But in *hence* narrative structures, the plot is developing, and episodes unfold in logical relation to their previous ones and to the whole (Frye, 2000). Let us consider a roguish, underclass character, of the type characteristic of a picaresque episodic story, in an *and-then* narrative structure who optimistically thinks that he is on the path to upward social mobility. He is naïve enough that he does not know that his life is disjointed and episodic and he dismisses the reality that for him there is no upward social mobility. His over-optimistic view of life does not let him see that he is not growing. His life, usually, consists of satirical and comical elements. He critiques political society as an outsider, with a humorous and exaggerating tone.

The character in the picaresque situation can gradually or suddenly become aware of himself and realise that his life has no meaning in its totality and he has been naive and over-optimistic about the growth and development in his life. This is the social situation that leads into activities with architectonic form of a picaresque novel. The moment of self-awareness will create a critical situation leading into a more conscious moment within the *perezhivanie-as-struggle*. Here, the problem is how to help such characters become self-aware of their own picaresque situation and undergo psychological progression and come out of their naivete. In such situations, turning the *and-then* narrative structure into *hence* narrative structure and bringing a meaning into the totality of the life would create a transformative shift in their characters (to learn more about the picaresque genre look at Friedman, 2022 and Garrido Ardila, 2015).

### Ressentiment Situations

In some situations, as Vasyliuk describes, self-change and environment-change in response to conflictual situations does not happen through a creative and self-affirmative process but instead through a reactionary and self-deceptive process. It cannot therefore properly be called a purely ‘successful’ perezhivanie-as-struggle because it also contains many self-destructive elements in the process of development. For example, imagine a situation where a person (or a group of people) has a desire to realize the value of political supremacy. But they are impotent and unable to reach political supremacy in competition with their rivals. The conflict they face then is whether to accept the reality and withdraw from political life, or to pursue the value and continue despite apparent impossibility. In a ressentiment situation, instead of reorganising their prisms in a way that would reaffirm their values (changing the reality of impotence, or reformulating the desire of supremacy such that it doesn’t cause them any problems if it not realised), they deceive themselves that they no longer harbour a desire for supremacy, and turn their will into a reaction against themselves (and their victorious rivals). Out of ressentiment and saying no to their own (and their rivals’) values, the ‘man of ressentiment’ should revaluate their own values and transform them in a way that would express condemnation of the rivals’ values (Reginster, 1997). But condemnation of their rivals’ values is also condemnation of their own values.

In a ressentiment situation the character develops while simultaneously losing his own integrity because of losing his truthfulness and self-honesty. The character of a ‘man of ressentiment’ is torn by the tension between his desire to live a life that he values (for example, “I want to become leader of the movement and take over the State!”) and his belief that he is unable to satisfy it. His reaction to his triumphant rivals in self-deceptive way (“Being leader and having the State is the problem!”) brings about division within him. However, his new values (For example, “let’s build leaderless organisations”) still tries to realize the very desire that he is desperately condemning. Hence, there is nothing creative in a ressentiment situation. The character makes no new meaning out of the situation except through negating the dominant code of values. Instead of creating a new from inside, the ‘man of ressentiment’ says no to “what is ‘outside’, what is ‘different’, what is ‘not itself’; and this NO is its creative deed” (Nietzsche, 2013). In the ressentiment situation one’s main motivation is not affirmation of his values hand in hand with creation of new ones. He only has the desire to deny older ones. In a ressentiment situation one is dealing with a tendency to split off all the negative aspects of their character and project them into outside world. The result is, on the one hand, the creation of a caricature of an oppressive, overpowered enemy, ruling the world and, on the other hand, a victimized self which is suffering from envy, passive aggression, hatred and repressed vengefulness.

## AutoEthnography and Perezhivanie

Autoethnography is one of the research methodologies that can be used to investigate perezhivanie. According to (Adams, Holman Jones, & Ellis, 2015):

Autoethnography is a qualitative research method that: (1) uses a researcher’s personal experience to describe and critique cultural beliefs, practices, and experiences; (2) acknowledges and values a researcher’s relationships with others; (3) uses deep and careful self-reflection—typically referred to as “reflexivity”—to name and interrogate the intersections between self and society, the particular and the general, the personal and the political; (4) Shows people in the process of figuring out what to do, how to live, and the meaning of their struggles; (5) balances intellectual and methodological rigor, emotion, and creativity; and (6) strives for social justice and to make life better.

I found the methodology of autoethnography to be compatible with the theoretical framework of ‘perezhivanie’ and useful in the case study of ‘becoming an environmentalist scholar activist’. It helped me to turn my everyday life into an ongoing and everlasting fieldwork. Every detail of my everyday experiences, from waking up and doing housework to going to the library, communicating with other academics, organizing meetings and events and then mobilizing people in marches, until going back to bed and having some dreams about my life turned into materials for the autoethnographic inquiry. So, my life-experiences within my family, friendship circles, organisations, communities, campaigns, marches, and other social spaces become worthy of examination. Focusing on a *particular* life rather than *general* information about a group of people, as one of the core ideals of autoethnography (Besio, 2020) helped me to integrate this method of inquiry into a process of self-analysis.

Autoethnography also helps us to identify and explore those unique moments of life that one can call *epiphanies* or those sudden moments of illumination that manifest themselves “in vulgarity of speech or of gesture or in a memorable phase of the mind itself” (Joyce, 2013, p180). In other words, those delicate and evanescent moments that, through their very triviality, suddenly express something extraordinary. How can the concept of ‘perezhivanie’ grasp those moments of life that you can “see a World in a Grain of Sand, and a Heaven in a Wild Flower, Hold Infinity in the palm of your hand and Eternity in an hour” (Blake, 2025, Auguries of Innocence)? I am referring to those irrational, intuitive, insightful moments that one’s life and character changes by being exposed to mundane details of everyday life. Those specific moments that one does not appear to create anything or resolve any critical situation but conceives a new meaning through a seemingly ordinary moment. Following James Joyce, researchers in the field of autoethnography have conceptualised these moments as epiphanic moments (for example see Bochner & Ellis, 1992; Adams, Holman Jones, & Ellis, 2015). These moments can range from aesthetic illumination to eureka breakthroughs in various fields of inquiry to a moral realization.

Ellis, Adams and Bochner observe that “When researchers do autoethnography, they retrospectively and selectively write about epiphanies that stem from, or are made possible by, being part of a culture and/or by possessing a particular cultural identity” (Ellis et al., 2011). So, through writing epiphanic moments, the autoethnographer foregrounds his personal experiences in research and writing. But autoethnography is not simply about epiphanic moments. It is also about processes of sense or meaning making (Bochner, 2001). By using perezhivanie as our theoretical framework we understand that we should not focus simply on the inner, subjective developmental processes of sense or meaning making and the way that a personal characteristic is developing. We should also focus on the interrelation between this inner developmental process and the changes that are occurring in the environment. Moreover, perezhivanie gives frame to autoethnographic research by guiding the investigation to distinguish various moments of character development and their corresponding activities. For example, without the concept of ‘perezhivanie-as-struggle’, autoethnographic investigation cannot explain the processes of radical shifts and changes in an individual’s life.

The toolbox of various methods of analysis in autoethnographic methodology is useful in analysing perezhivanie processes, specially, in my case, the *perezhivanie-as-struggle* of a scholar willing to become an environmental scholar-activist. This methodology is not only useful in analysis of ‘perezhivanie-as-struggle’ but also, as a self-reflexive and critical activity, facilitates the process and has psychotherapeutic effects. The experience of doing autoethnography, i.e., writing diaries, drilling down into my deeper emotions and reasons of my actions, expressing my desires in artistic forms, such as poetry or storytelling, has helped me to work through difficult moments of my life. These include the process of migration to England, joining new communities, and divorcing my partner, after 13 years of being together. The self-reflexive character of this methodology was helpful in rearticulation of moral and political lines of thought.

The practice of doing autoethnography generates stories and rich descriptions of social situations that are helpful in doing the aesthetic investigation. For example, the collection of diaries, as well as detailed description of epiphanic events provided me the texts to analyse the content of my *perezhivanie-as-struggles* and distinguish the architectonic forms of each situation that was leading into reconstruction of my personality as a scholar-activist.

Conducting an autoethnographic inquiry starts by ‘moving inward’ and situating ourselves in our stories. Whether through mundane details of everyday life, epiphanic moments, or critical situations of *perezhivanie-as-struggle*. The inquiry contains writing down thick narration of such details, moments and situations and analysing them to find recurring themes, concepts, plots, or anything else that would give us some knowledge about the character of sensibilities of ourselves. During the moments of ‘moving inward’, I was also learning about various literary genres and styles to figure out which ones are expressing the aesthetic form of my stories. Autoethnography also includes ‘moving outward’ and discussing the topic of research with those scholars (and in my case those scholars and activists) who have the same research interests to find more clues or gaps about the topic. These processes of moving inward and outward and writing about the details of everyday life helped me to observe and analyse the process of creation of my new characteristics and the aesthetic forms of those new creations. For this reason, autoethnography, through producing thick texts, is also a useful methodology to analyse the aesthetic aspects of an individual’s experience.

In a nutshell, autoethnography equips the researcher with a wide range of methods helpful to investigate the details and nuances of psychological and aesthetic aspects of becoming a scholar-activist and it is compatible with the theoretical framework of ‘perezhivanie’ that has been put forth in this article.

### Autoethnographic Corpus and Analytical Procedure

For this autoethnographic inquiry I mainly used the *autoethnographic diaries* that I produced between September 2021 to September 2025. I have the habit of writing my diaries on a weekly basis. Apart from that, because of my PhD research, I wrote weekly diaries with special focus on memories of conversations with activists, speculations on political events, and my feelings and thoughts on my process of becoming scholar-activist. Fieldnotes and personal communications are included in the *autoethnographic diaries*, but apart from that I used photos and videos that I took from social movements’ events as a tool to remember epiphanic moments. Epiphanic moments as well as moments that expressed the critical situation and the onset of perezhivanie-as-struggle were selected and further analysed during the autoethnographic inquiry. These analyses, hand in hand with learning more about aesthetic theories and literary genres, led into development of absurd, picaresque, and ressentiment themes, which later been applied to re-interpret the diaries.

I wrote most of the diaries in Farsi. The excerpts that have been demonstrated in this paper are all translated into English. I have anonymized the excerpts by changing the real names and gender of the people been mentioned, deleted the name of organisations, with the exception of Co-Operation Manchester, which their main organisers have kindly given me the consent to use the name of the organisation in this research.

## A Brief AutoEthnographic Account

In this part I will briefly describe the autoethnographic findings as well as an outline of the aesthetic analysis of the *perezhivanie-as-struggle*. The findings depict the story of a character that is dealing with multiple senses of alienation and isolation, struggling with a fragmented life, and working over resentiments that are growing out of a politically weak position in society as being communist and exiled. It is the story of an exiled scholar who wants to become an environmental activist whose activities are linked with academia, working-class communities, environmentalists and Kurdish and Iranian exiled/diasporic communities. His *perezhivanie-as-struggle* creates new ethical values, such as the necessity of synthesizing different genres of activities, overcoming specialisations of skills, knowledge and life-domains, educating sensibilities as well as reasons, and struggling for self-determination. In what follows we will examine these findings in more detail.

### From a Clinical Psychologist to an Environmental Scholar-activist

Since September 2021 when I started my PhD at the university of Manchester, as part of my research project to become a scholar-activist I tried to build my connections in various networks. First in the Farsi speaking exiled communities, with the hope of finding like-minded people who are politically active. Second, among the students and scholars in academic spaces, with the hope to learn more about environmentalism, communism, scholar-activism and solidarity between social movements. Third, among the activists and community organisers with the hope of learning more about existing movements and grassroot initiatives. Before that, I was a clinical psychologist spending most of my time working in clinical settings. I didn’t know much about scholar-activism.

The emergence of the *Jina Uprising* or *Woman*,* Life*,* Freedom* movement in Iran, was a turning point for the Kurdish and Iranian communities in Manchester. Various communities and political networks began to organise demonstrations and political events. I also joined these events and searched for link-minded people with Leftist ideologies to become friends and work with. After a while I found some isolated Kurdish and Iranian communists and feminists who were living in the South of Manchester. They had almost no connection with the political community of Manchester and were experiencing a strong sense of insecurity, fear and suspicion of newcomers. This section of my diary after visiting an Iranian communist family in south of Manchester shows the anxiety and protectiveness:

After the Palestine demo in the St. Peter Square, I accepted the invitation of Asghar [pseudo name] and his partner to go their house for dinner and chat. When we arrived, other friends were also there … We were having a beer, and the atmosphere was very calm, happy and friendly. I started to ask about the possibility of working with [a political organsiation in Manchester] to receive support from them. Asghar looked at me suspiciously. ‘I don’t know them’ said Asghar. ‘who knows what kind of dodgy organisation is funding them’. I told them that I know which foundations are providing their fund and it is transparent on the website of … [the name of the organisation]. After this exchange, the atmosphere of the house changed. Everybody became silent and nervous. Asghar, with a tone which was more cautious and a bit mysterious, said that “you might be a newbie in Manchester and don’t know much about what is going on. Some of these foundations are linked to security agencies”. I didn’t respond to Asghar. But I felt that they might have seen the expression of surprise in my face. “Sohrab, listen to me!” said Asghar “we should keep the doors and windows of our house shut! It is a stormy winter outside!” (*autoethnographic diaries*, 06/08/2022).

I began to understand that to become part of an exiled diasporic community of political activists I need to learn their isolationist and suspicious attitude and focus on keeping ‘our house shut’ rather than developing the network and connections of the house with the social and political life in Manchester. But this was a restricting situation. I found this to be a problem in the exiled diasporic communities rather than a characteristic that needs to be reproduced. This experience taught me that breaking the chain of isolation from local politics, overcoming difficulties in establishing links with other communities and working over the dominance of the anxious/fearful sensibility are among my pedagogical tasks. I remember an important conversation with one of my friends in Iran about the situation of diasporic communities of Iranian political activists: “creation of isolated and closed political sects in diaspora could be an adaptive strategy to maintain a political line or tradition that is heavily under threat. It could also be the very reason for a political tradition to die because of not opening itself to new ideas and people. So, you should be very careful how you are going to engage with this problem” (personal communication, 13/08/2022).

Close collaboration with these friendship circles in organising public events, protests, demonstrations and political meetings, was simultaneous with participation in local activities of environmentalist groups. Also, I was trying to enrich my theoretical understanding of environmentalism through attending and organising seminars of *Green Research in Education Group* (GRiEG) in Manchester Institute of Education. One of the main characteristics of these environmentalist groups was their extreme suspicion about vertical organising, party politics and the State. This sensibility objectified itself, very clearly, in the protest camp that some environmentalist groups made in the ‘Ryebank Field’ to save this green land and its biodiversity from destruction by construction activities. Saunders and Price’s explanation of protest camps’ objectives (2009) was useful in understanding the objectives of ‘Ryebank Field’ Protest camp: (1) facilitate direct action to prevent climate catastrophe in a given area (2) performing an alternative mode of living (based on ecological and anarchist principles) (3) building quasi-formal educational programs (Niesz et al., 2018) by holding open workshops and (4) building an environmental social movement capable of influencing policies of banking system and big corporates. The protest camp in the Ryebank Field was practicing a utopia building project, with the ambition of making a leaderless, horizontal community of activists. Although the ‘culture of security’ (using pseudonyms, turning off mobile phones in meetings, using encrypted communication apps, etc.) was very widespread among these activists, they were very open to newcomers, especially students, and were happy to mix with activists with different ethnic backgrounds. However, they were feeling and reflecting on their problem of isolation from working class communities and organised workers in trade unions. For example this part of the autoethnographic diary clearly expresses the situation:

After the meeting between members of the protest camp and other environmentalist networks that were coming from … and …, we collectively washed the dishes and cleaned the camp. Me and Saffron [pseudo name] turned into a pair and helped each other in cleaning tasks. I started the conversation by asking her about one of the main items of today’s meeting agenda, which was limited participation of local community in maintaining the protest camp. She said that there are a few retired members of the community who, sometimes, come and stay in the camp for a few hours. “They all come from a middle-class background” she said “and I don’t know how can we attract more working-class people? People from other areas of Manchester?”. “Do you have any connection to trade unions?” I asked. “Some of our friends set up the [name of the organisation] to link up with trade unions. But it is not really working” she said (*autoethnographic diaries*, 15/06/2022).

As a scholar-activist I was in an environment where I could think about these problems in academic terms and settings. Could I bring my academic knowledge into useful dialogue with these social movements? Scholars in the GRiEG were promoting a concept of eco-socialism that provides a coherent conceptualisation of internal relation between ecological processes and social projects that goes beyond the Nature/Society dichotomy (Moore, 2015). Here is an account of GRiEG’s environmentalism that I found in conversation with one of the GRiEG members: “I think about that [environmental issues] holistically. I don’t think you can separate capitalism from the crisis of the environment. That is all connected. And the pandemic is just a particular response of the nature to a desperate reduction in biodiversity, which is caused by people like [Jair] Bolsonaro, people in the Amazon chopping down trees [and] through the monocultural industrialization in large parts of the Far East, etc, etc, etc. So, I never saw the green thing as being separate from the social equality issue and the problem of doing something with capitalism. What I think the green agenda does is to work with a new realization that we’re all fu**ed if we don’t do something about this”.

GRiEG’s conception of environmentalist scholar-activism motivated me to take the problem of alliance between climate and labour political campaigns and industrial actions more seriously. However, in the field, I observed not only the difficulties of people in the protest camp to connect with the organised labour force and working-class communities, but also the difficulties of academics and their activities to connect with the movements of the working class communities and environmentalists. The language of academic writing, the setting of academic seminars, and exclusivity of events and conferences are creating a gap between academics and movements of people in their communities. The rift between the polite education of academia and popular education among the people was another problem that I experienced.

In other words I was experiencing a fragmented life. Isolated Kurdish and Iranian diasporic/exile communities. Environmentalists having difficulties in linking up with working class communities. And academic networks with exclusive language that is unable to communicate with the above people. These different fragments of my life couldn’t connect with each other to make a coherent whole, and, in Vygotsky’s terms, that was my ‘social situation of development’:

It might be true that the experience of migration to Manchester made me more sensitive about the problem of overcoming isolation. It might be true that I am prone to observe the social phenomena through my biased mindset which is magnifying the fragmentations. I am aware of my desire for mixing and infusion. But this doesn’t invalidate the significance of the problems that I have observed among the Kurdish/Iranian exile communities, environmentalists and the academic communities. The particular positionality of me, as a migrant Kurdish/Iranian scholar-activist who is learning to become environmentalist is revealing these problems to me. I think I should take them serious and work toward solving them (*autoethnographic inquiries*, 23/03/2023).

On the theoretical side of the problem I began to deal with the questions related to organisational infrastructures, political ideologies and narratives that can bring together grassroot but fragmented forces into cooperation with each other. The experience of living in Manchester taught me that working-class communities are dealing with multiple problems, such as food poverty, rent rise, taking care of refugees, rise of far-right racism, unemployment, etc. Hence, it is important to consider the multiplicity of organisations that can tackle these problems. Such as food banks and food justice networks, tenants unions, refugee support groups, anti-fascist networks, co-ops and community-interest businesses. So, I began to learn about political and educational theories that could help me and other activists in fostering solidarity and cooperation between all these initiatives and the environmentalist campaigns and the academic communities. An epiphanic moment in my life which I described in my autoethnographic diaries happened when in a meeting with some environmental activists and their families we collectively wrote about the needs in our communities:

… we were all looking at the big piece of brownish paper on the table while reflecting, in silence, on the problems and needs in our community. After a while, everybody were busy with writing all sorts of things on the paper. From very concrete needs, such as “bread and water” to very abstract needs such as “public ownership” or “labour self-management”. After the urge for writing the needs calmed down, I asked them “what sort of organisation can respond to all these needs in our community?”. I paused with an anticipation of response. But after a few seconds I completed my question “if not the State?”. I hoped that somebody would negate my suggestion of “the State” and initiate a response. “I think we should learn a lot from the experience of Cooperation Jackson”, said Saffron. Cooperation Jackson. This is now the main source of inspiration for us. (*autoethnographic diaries*, 28/08/2023) (Fig. 2)

**Fig. 2:**
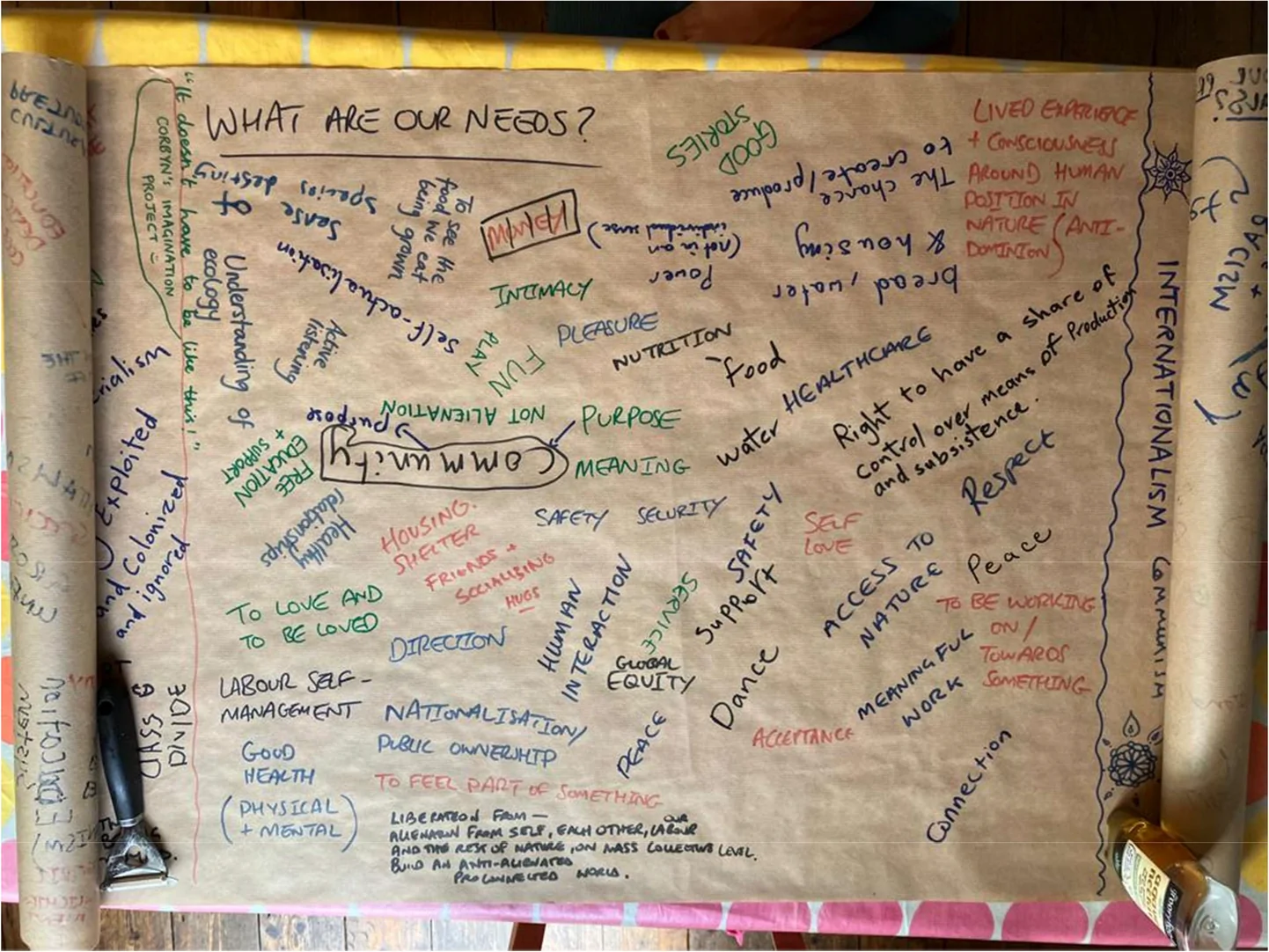
Photo of the reflection on our community’s needs, mentioned in the autoethnographic diary 28/08/2023)

The result of conversations and collective discussions came into fruition through the establishment of Co-Operation Manchester. This initiative began to facilitate socializing between various organisations, helping them to link up with each other. The process of establishing Co-Operation Manchester in the early months of 2024 led to raise in the awareness of working-class initiatives in the south of Manchester about each other’s existence and their current activities. On the day of the formal launch party of the Co-Operation Manchester more than 20 organisations (including local trade union branches, community organisations, co-ops, environmentalist businesses and campaigns, refugee support groups, tenant unions, and international solidarity networks) signed up to cooperate within the platform of Co-Operation Manchester. After the launch party Co-Operation Manchester began to organise popular assemblies in South of Manchester. These assemblies not only were acting as spaces for communication and deliberation between various organisations and ordinary people to decide about new local projects, but also were helpful in organising popular education projects.

Initiating and supporting Co-Operation Manchester on the personal level was a response to my experience of a fragmented life. Engagement with Co-Operation Manchester was an attempt to make a coherent whole out of different, isolated pieces of my life and link up academic, environmentalist and working class activities. Life as a clinical psychologist with limited political activity became meaningless and I had to make new meanings, ethical principles and practices. The whole time, the scholar-activist PhD research and the conversation with my supervisor, Julian Williams, was leading this *perezhivanie-as-struggle*: “Your scholarship is a dialectic of collective research, learning and teaching that is done *by* the activists *fo*r the activists”.

This perezhivanie-as-struggle was not without failures and difficulties. The most important one was the failure in bringing the exiled community of Kurdish and Iranians out of their isolation and sectarian mindset. The more I was getting involved with the network around Co-Operation Manchester, the more I received suspicious comments from Iranian activists who were solely focused on issues related to Iran. Some people decided to cut their relationships with me, which at the end led into complete ostracization from a specific community of exiled Iranian political activists. I wrote about this experience in one section of my autoethnographic diaries:

The online meeting with … [name of the group], which supposed to be a regular member meeting with usual biweekly agenda, turned into a very emotional trial of my deeds in the last two years. Each member of the … [name of the group] was taking the mic, one by one, and beginning a 10-minute speech about me and things that I did wrong. One individual raised a question about my ESRC fund claiming that ESRC studentships are made by MI5 to operate espionage projects among the political diasporas. Someone else called me ‘an opportunist who works with organisations who have interests other than serving people’. Another one claimed that I ‘betrayed my community’ by working with English people on issues that are not related to Iran, and someone else lamented that I created a lot of stress in the group because of making all these ‘connections with strangers’ and doing ‘parallel political works’. At the end of the session, it was very clear for me that I don’t have a place in this group anymore. They were ostracizing me. (*autoethnographic diaries*, 25/11/2023)

### The Content of the Perezhivanie-as-struggle

In a self-reflective way, two months after the beginning of the activities of ‘Co-Operation Manchester’, I asked myself (and discussed it with my friends and comrades) what are the meanings of my new activities? What are the new ethical values that I am following (or making)? If one considers the new activities (for example establishing Co-Operation Manchester) as the *form* of my perezhivanie-as-struggle, I distilled the *content* of it through examining my own activities. I collected all the answers and reflected on them in my autoethnographic diaries. Later on I selected those propositions that were explaining my activities as scholar-activist during the period of establishing Co-Operation Manchester:

**1- “**Scholar-activism is not restricted to a specific discourse or genre of activism, distinct from other ones. It is an eclectic activity in essence and should combine and synthesize all the concepts and projects that are developing in social movements.**”**

This is a response to the experience of fragmentation of struggles and isolation of various aspects of life from each other. A response to the precarity and episodic nature of my activities as an exiled scholar.

**2- “**A scholar-activist should strive in the direction of developing and realizing all the powers, in himself, that are general to human beings, and also the powers that are characteristics of his unique individuality. This strive, definitely, needs to overcome the divisions of labour and traditional specialization of manual/mental labour, popular/academic education, revolutionary/real politics, etc”.

This ethical proposition stems from a romantic approach to science (Cole et al., 2006) and a socialist strive to make an all-round developed human being capable of taking control of his own social life (Vygotsky, 1994). A response to limitations of academic forms of education that excludes ordinary people from learning and producing knowledge. And finally, a response to exclusivity of forms of politics (especially among the environmentalists) that are unable to connect to working-class communities and their everyday problems.

**3- “**A scholar-activist should educate not only reason but also sensibilities. Revolutionary theories not only should touch the heads of people, but also their hearts. Otherwise, people are not inclined to act based on revolutionary theories”.

This is a response to the problem of exiled/diasporic communities, such as Kurdish and Iranian communities that are suffering from difficulties in developing trust and are fearful of newcomers and other communities. The education of inclinations is as important as the education of cognitive reasoning.

**4- “**A scholar-activist should constantly strive for self-determination. i.e., to realize the powers of the communities that the scholar-activist belongs to and act based on their internal laws, without any external constraints. This is the strive for freedom”.

This is a response to the problems of exiled/diasporic communities, such as Kurdish communities, that are struggling for self-determination of their communities in their homeland. Kurds as oppressed people convey this ethical proposition.

### The Form of Perezhivanie-as-struggle

Was it possible for me to realise the above *content* in the forms of my scholar-activism? Were the social conditions in favor of the objectives that I wanted to realise? In the early months of 2024, I began to ask these questions. Is not scholar-activism, at least in the context of Manchester, restricted by objectives, rules and regulations of the available funds that have been allocated by different organisations? The experience of becoming scholar-activist showed me that most of the funds that are enabling scholar-activism come from above’ organisations in the State departments and charitable foundations. These funds will be given under certain conditions that require the scholar-activist to reproduce a range of ideas and concepts that logically cannot undermine the fundamental norms and laws of the bourgeoise society that provides such funds. There is a cap on the level of consciousness of scholar-activists that eventually contradicts the environmentalist and anti-capitalist principles that she is following. In 2024 I began to experience the conflict between the revolutionary content of scholar-activism and the form of practice that I needed to adapt. I was feeling a deep frustration while I was writing these sentences in my diary:

most of the time, I feel that I am in an absurd novel. What I am doing is far from a clear contribution to the anti-capitalist movements. Most of the time I feel that my activities and revolutionary desires result in nothing! Organisers and revolutionary people seem very confused and weak. A few months ago, a comrade told the most minimal absurd story that reflects our situation very well: *We are the Left. Yes. But we are left [abandoned]!* (*autoethnographic diaries*,* 12/03/2024*).

In addition to the absurdities of becoming communist and environmentalist scholar-activist, the precarity of the political projects and lack of any meaning in the totality of local projects was creating an environment ready for growth of a naive character. The experience of being within the “pool of activists” in South Manchester showed me that the young, energetic and mostly student groups of activists are happily becoming volunteer in many social and political events: from international solidarity demonstrations, feminist campaigns, and environmentalist public discussions, to anti-deportation projects, refugee support groups, community meals and socialising events. By joining whatsapp groups and other social networks of activists, very soon I found myself within a multiplicity of events and projects that were demanding activists to join and help. It was very difficult for me to prioritize different activities and understand how these different activities are linked to each other. After a while, I figured out that my life does not make any *coherent totality* and I am just following events one after another. Initiating Co-Operation Manchester and learning about Solidarity Economy as the political strategy which gives a coherent meaning to the fragmented activities were among the efforts to overcome the picaresque and episodic nature of rolling between various projects and movements.

The weakness of the communist and environmentalist movements (and in general, the weakness of the Left) has led to the development of all sorts of ideologies that denounce political tactics and strategies for gaining power. The weak, resentful forces, instead of recovering from the previous defeats, began to attack the very idea of victorious powerfulness, while praising and promoting different manifestations of defeatism. Despising strategies of power grabbing and disciplined self-organisation leads into despising virtues and ethical values that growth of which is necessary for an all-round developed revolutionary.

## Final Comments

Perezhinvanie-as-struggle as a concept within the cluster of Perezhivanie concepts that expresses the radical changes and ruptures in the development of personality is a useful device for educational purposes. This article attempted to clarify and enrich this concept with the Bakhtinian aesthetic theory that shows how an artistic creation unifies the cognitive knowledge and ethical values as the content of a work of art that expresses itself in given material form.

This study suggests that using autoethnographic methodology in dialogue with aesthetic theories facilitates the process of analysing a *perezhivanie-as-struggle* by turning it into a story, distilling its content (the ethical propositions that are responding to perceived realities) and examining its forms.

Theoretically, this paper proposes the significance of concepts that are useful to explore the social situations of development that are not straightforward situations of growth. People, instead of a linear growth, might experience many hopeful progressions and hopeless regressions. Therefore, concepts such as absurd, picaresque or ressentiment situations might be useful for exploring the regressive moments in any individual’s developmental path.

Pedagogically, any educational project that has the goal of training scholar-activists might benefit from identification of failed perezhivanie-as-struggles or regressive elements in those periods. Educational projects can benefit from self-reflective tools in the toolbox of autoethnographic methodology and help the scholar-activists to narrate their own life-stories and learn about the challenges and problems that they need to overcome in their process of identity formation.

## Data Availability

No datasets were generated or analysed during the current study.

## Footnotes

1 “You should love peace as the means to new wars. And the short peace more than the long one.” (*Thus Spoke Zarathustra*, On war and warriors, p33).

2 the fictional protagonist of the 1866 novel Crime and Punishment by Fyodor Dostoyevsky.
